# Sensor Selection and State Estimation for Unobservable and Non-Linear System Models

**DOI:** 10.3390/s21227492

**Published:** 2021-11-11

**Authors:** Thijs Devos, Matteo Kirchner, Jan Croes, Wim Desmet, Frank Naets

**Affiliations:** 1 LMSD Research Group, Department of Mechanical Engineering, KU Leuven, Celestijnenlaan 300, 3001 Leuven, Belgium; matteo.kirchner@kuleuven.be (M.K.); jan.croes@kuleuven.be (J.C.); wim.desmet@kuleuven.be (W.D.); frank.naets@kuleuven.be (F.N.); 2 DMMS Core Lab, Flanders Make, Gaston Geenslaan 8, 3001 Leuven, Belgium

**Keywords:** extended Kalman filter, state estimation, sensor selection, observability, non-linear models

## Abstract

To comply with the increasing complexity of new mechatronic systems and stricter safety regulations, advanced estimation algorithms are currently undergoing a transformation towards higher model complexity. However, more complex models often face issues regarding the observability and computational effort needed. Moreover, sensor selection is often still conducted pragmatically based on experience and convenience, whereas a more cost-effective approach would be to evaluate the sensor performance based on its effective estimation performance. In this work, a novel estimation and sensor selection approach is presented that is able to stabilise the estimator Riccati equation for unobservable and non-linear system models. This is possible when estimators only target some specific quantities of interest that do not necessarily depend on all system states. An Extended Kalman Filter-based estimation framework is proposed where the Riccati equation is projected onto an observable subspace based on a Singular Value Decomposition (SVD) of the Kalman observability matrix. Furthermore, a sensor selection methodology is proposed, which ranks the possible sensors according to their estimation performance, as evaluated by the error covariance of the quantities of interest. This allows evaluating the performance of a sensor set without the need for costly test campaigns. Finally, the proposed methods are evaluated on a numerical example, as well as an automotive experimental validation case.

## 1. Introduction

Nowadays, many new mechatronic systems become available on the market, designed to perform tasks with increasing complexity while ensuring machine/operator safety. As a result of this increase in complexity, advanced controller schemes are being developed that introduce the need for accurate information on the dynamics of these systems. However, in many applications, this information cannot be directly measured because either no sensor exists that is capable of measuring the quantity of interest or the available sensors are expensive or impractical to implement [1]. This has led to the development of various virtual sensing techniques that aim at obtaining the dynamic information of a mechatronic system indirectly through estimations based on simple measurements  [2]. This methodology has been investigated for use in a wide range of applications [3,4,5,6].

As a result of increased computational power, these virtual sensing schemes have gained significant traction for the analysis of structural [7] and general mechanical systems [8,9] in recent years. Driven by the need for more accurate system information, model complexity has significantly increased up to flexible multibody-related formulations [10,11,12]. However, this comes at the cost of increased computational effort and additional issues towards observability and thus estimator stability.

When model complexity is increased, observability requirements can become challenging as extra model degrees of freedom require additional sensor data to ensure full observability [13]. For example, in tire force estimation applications, vehicle position states are typically unobservable when no GPS measurement is present, although these measurements do not contribute significantly to the tire force estimation performance [1,14]. Furthermore, the non-linear governing equations create additional challenges to evaluate observability globally and/or locally [15,16]. Additionally, some virtual sensing applications feature models with lots of states (e.g., for meteorology or oceanography typically $>10^{6}$ [6]), of which only a few significantly contribute to the estimator performance. This indicates that there is a need for an approach to deal with unobservable, and thus unstable, estimators given the interest in only a few specific quantities.

One of the approaches proposed in the literature to deal with large system models is to reduce the order of the estimator to decrease its computational efforts and set fewer constraints towards observability. Two main approaches to reduce the order of an estimator have been presented in the literature. The first approach involves reducing the model if this model can be approximated sufficiently well by a subset of modes [17,18,19]. However, this approach is highly model specific and is therefore not suited for all applications. The second approach involves reducing the order of the Kalman filter by transforming the Riccati equation associated with the filter [1,6]. Yonezawa et al. [20] have presented an approach for unobservable system states to be asymptotically observable, assuming that a dynamic link is present between the unobservable and the observable states. This approach has proven to be very effective when considering unknown parameters or constant bias errors. However, this approach only works when a dynamic coupling is present between unobservable and observable states, which is, in a lot of applications, rarely the case for all system states, especially when dealing with increasing model complexity.

For application cases with decoupled state dynamics, such as unknown accelerometer biases and unknown parameters, the Schmidt–Kalman filter can be deployed [21]. The covariances corresponding to the augmented states are not taken into account in the update steps as these states are typically not measurable and dynamically completely decoupled from the other states. This makes it impossible to observe these states; hence, they are simply omitted from the update step. When many of these states are present, this technique can drastically improve the computational effort as only the observable state covariances are being evaluated [22]. On the other hand, the Schmidt–Kalman filter approach can only be defined for completely decoupled augmented states.

Finally, sensor selection is a necessary step when deploying any estimation framework. A common approach is trial-and-error as sensors typically have to be bought and deployed before information can be acquired on their performance. However, previous research has already investigated some more cost-effective approaches that vary widely from equation-based modeling approaches [23] to machine learning methods [24]. Commonly proposed objective functions for model-based sensor selection algorithms are the Fisher Information Matrix (FIM) [25] or the error covariance matrix of the estimator [26], evaluated using the Riccati Equation (ARE) or the Lyapunov Equation [27]. However, all of these methods still rely heavily on sensor information being available that contains information on the sensor performance, which usually requires highly expensive test campaigns for which sensors have to be acquired.

As an alternative to the above-described schemes, this work presents a novel approach to stabilise a non-observable, non-linear estimator for both full and partially decoupled system dynamics given that only specific quantities of interests are targeted and proposes a new sensor selection strategy that is able to evaluate sensor set performances before acquiring them. Firstly, an estimator setup is proposed based on a set of non-linear governing equations for both the system model, measurements and quantities of interest, described in Section 2. Furthermore, a projection of the estimator covariance equations on an observable subspace is proposed in Section 3, which makes it possible to stabilise the estimator covariances during operation. The projection explained in Section 3.2 is based on the singular value decomposition of the linear Kalman observability matrix. Furthermore, the projected Kalman filter equations presented in Section 3.3 can stabilise the unobservable state covariances although these states remain unobservable. Hence, their resulting covariances should be interpreted with care. Additionally, a sensor selection methodology is proposed, which ranks the sensors based on their estimation performance such that the user can obtain an overview of the best sensors to choose from, as defined in Section 4. This has the great advantage that a prior analysis of the sensor performance can be carried out that eliminates the need to acquire sensors beforehand and organise costly test campaigns. Finally, the work is validated in two different cases, namely an academic example explained in Section 5.1 where the potential of the method is shown and a full experimental validation case explained in Section 5.2.

## 2. Model Definition and Overall Estimator Setup

In this work, a non-linear model will be considered to govern the dynamics of a physical system, the measurements and some predefined quantities of interest. These non-linear models are furthermore linearised and discretised in an Extended Kalman Filter [13] based the estimation framework. This section describes the implementation of the general estimator equations.

### 2.1. Definition of Model, Measurement and Quantities of Interest Equations

The non-linear equations considered in this work can be divided into system, measurements and quantities of interest equations and are introduced as:

(1) $$\begin{array}{ccc} \mathrm{Model}:\; & & \dot{x}=f\left(x,t\right) \end{array}$$

(2) $$\begin{array}{ccc} \mathrm{Measurements}:\; & & y=h(x,t) \end{array}$$

(3) $$\begin{array}{ccc} \mathrm{Quantities}\;\mathrm{of}\;\mathrm{Interest}:\; & & y_{vs}=g\left(x,t\right) \end{array}$$

where x is the state vector and *t* is the time. The vector $\dot{x}$ describes the time derivative of the states, and the vectors y and $y_{\mathrm{vs}}$ are the measurements and quantities of interest, respectively.

In this work, an estimation framework is set up based on the discrete extended Kalman Filter for which an explicit relation is necessary. Therefore, these continuous non-linear equations have to be linearised and discretised. This is discussed in Section 2.2.

### 2.2. Linearisation and Discretisation of the Governing Equations

To obtain the linear and discrete Jacobian matrices used for the estimator evaluation, the non-linear equations are linearised and discretised. The Jacobians are obtained by performing a linearisation around the a priori estimation point $\left(x_{k|k},t_{k}\right)$ by:

(4) $$\begin{array}{ccc} F_{c,k} & = & \frac{\partial f(x,t)}{\partial x}|{}_{x_{k|k},t_{k}} \end{array}$$

Furthermore, the rest of the linearised Jacobians are calculated accordingly:

(5) $$\begin{array}{ccc} H_{k} & = & \frac{\partial h(x,t)}{\partial x}|{}_{x_{k|k+1},t_{k}} \end{array}$$

(6) $$\begin{array}{ccc} G_{vs,k} & = & \frac{\partial g(x,t)}{\partial x}|{}_{x_{k+1|k+1},t_{k}} \end{array}$$

Important to note is that any linearisation scheme can be deployed, which suits the application of the estimator. In this work, the linear continuous time Jacobians are obtained using forward differencing [28]:

(7) $$F_{c,k}=\frac{\partial f(x,t)}{\partial x}{|}_{x_{k|k},t_{k}}\approx \frac{f\left(x_{k|k}+\epsilon ,t_{k}\right)-f\left(x_{k|k},t_{k}\right)}{\epsilon }$$

The next step is to derive the discrete system Jacobian used for the integration of the system equations and evaluation of the Kalman filter covariance equations. Again, any type of discretisation scheme could be employed here, which best suits the application of the estimator. In this work, we chose to use a different discretisation method for the integration of the system equations and the derivation of the discretised sytem Jacobian for the Kalman covariance equations. This is conducted because it is not always practical to use the same discretisation scheme as this can lead to numerically ill-conditioned systems, for example, when dealing with a set of stiff equations [29].

For the derivation of the discretised system Jacobian used in the Kalman covariance equations, the exponential discretisation scheme [30] is used:

(8) $$F_{k}=e{}^{F_{c,k}\Delta t}$$

where $F_{c,k}$ is the continuous time system Jacobian obtained from Equation (7) and Δt is the timestep. The obtained discrete Jacobian is furthermore used in the EKF covariance equations.

For the integration of the system equations, a solver that is especially suited for stiff equations from the Matlab *ode-suite* is employed [31].

### 2.3. Overview of the Extended Kalman Filter Framework

In this work, the Extended Kalman Filter [13] is chosen for the estimation of the dynamic system information. This filter is an extension of the regular Kalman filter, making it applicable to non-linear equations. Using the previously calculated linearised Jacobians, the general estimator equations can be defined. In this process, the propagation of the states is conducted using the earlier defined non-linear equations, but the propagation of the covariance equations is conducted using the linearised matrices. The EKF-algorithm consists of three main steps, which are prediction, correction and update.

*Prediction*:

(9) $$\begin{array}{ccc} & x_{k|k+1}=f\left(x_{k|k},t_{k}\right) & \end{array}$$

(10) $$\begin{array}{ccc} & y_{k+1}=h\left(x_{k|k+1},t_{k}\right) & \end{array}$$

(11) $$\begin{array}{ccc} & P_{k|k+1}=F_{k}P_{k|k}F_{k}^{T}+Q_{d} & \end{array}$$

*Correction*:

(12) $$\begin{array}{cc} S_{k}=H_{k}P_{k|k+1}H_{k}^{T}+R & \end{array}$$

(13) $$\begin{array}{cc} K_{k}=P_{k|k+1}H_{k}^{T}S_{k}^{-1} & \end{array}$$

*Update*:

(14) $$\begin{array}{ccc} & x_{k+1|k+1}=x_{k|k+1}+K_{k}\left(y_{m,k+1}-y_{k+1}\right) & \end{array}$$

(15) $$\begin{array}{cc} & P_{k+1|k+1}=\left(I-K_{k}H_{k}\right)P_{k|k+1} \end{array}$$

As the quantities of interest are governed by non-linear equations such that $y_{vs}=g\left(x,t\right)$ in this work, a first-order Taylor expansion of the quantities of interest is considered around the previously calculated state configuration $\left(x_{k+1|k+1},t_{k}\right)$ to derive their covariances:

(16) $$\begin{array}{ccc} & y_{vs}\approx g\left(x_{k+1|k+1},t_{k}\right)+G_{vs,k}\left(x-x_{k+1|k+1}\right)+K_{vs,k}\left(t-t_{k}\right) & \end{array}$$

where $G_{vs,k}=\frac{\partial g(x,t)}{\partial x}{|}_{x_{k+1|k+1}}$ and $K_{vs,k}=\frac{\partial g(x,t)}{\partial t}{|}_{t_{k}}$ are the Jacobians of the quantities of interest according to Equation (6). When evaluating Equation (16) around the current configuration point $\left(x_{k+1|k+1},t_{k}\right)$ while assuming that the function g is approximately affine in this region, it can be stated that, up to a first-order approximation of the Taylor series, the quantities of interest will be a stochastic variable with the following mean and covariance [32,33,34]:

(17) $$\begin{array}{ccc} & y_{vs,k+1}=g\left(x_{k+1|k+1},t_{k}\right) & \end{array}$$

(18) $$\begin{array}{ccc} & P_{vs,k+1}=G_{vs,k}P_{k+1|k+1}{\left(G_{vs,k}\right)}^{T} & \end{array}$$

Since in most applications only a selected number of quantities of interest are targeted by the estimator, this works aims at focusing the estimator performance on these specific quantities of interest while keeping the estimator stable.

In this work, only a few predefined quantities of interest are estimated, which are evaluated using their error covariance. However, the covariances of the states themselves are important for the stability of the filter as the estimator is only stable when all states are at least detectable [23]. This is unwanted when a certain sensor does not improve the estimation quality of the quantities of interest but is required to make all estimator states observable, in which case one would like to omit this sensor from the estimator. Therefore, this work aims at solving the following major difficulties:

1. Stabilisation of the EKF for non-observable system states;
2. Selection of the relevant sensors for the given quantities of interest.

Both of these approaches will be discussed in the next two sections of this work.

## 3. Stabilisation of the Extended Kalman Filter for Unobservable System States

When employing estimators, it is well known that observability is an important topic [1,35] as it determines the stability of the filter. Because in this work only some predefined quantities of interest are considered, it might occur that some sensors do not have any effect on the estimation performance for these specific quantities of interest. They can however cause some states to be unobservable, which can lead to stability issues of the estimator algorithm. In this work, a projection is proposed to stabilise the unobservable state covariances given that only some predefined quantities of interest are targeted. Important to note here is that only the stability issues are being tackled. The targeted states remain unobservable, but the estimator error covariances will be stable. The derivation of the observable subspace consists of three main steps:

1. Generation of the total observability matrix $O_{tot}$ based on training data;
2. Calculation of the observable subspace basis $V_{o}$;
3. Transformation of the Kalman covariance equations.

These steps are discussed in the following subsections.

### 3.1. Observability Analysis of the Extended Kalman Filter

The observability investigation starts with the computation of an observability criterion. In this work, the linear Kalman observability matrix [36] is used, which is based on the Jacobians $F_{k}$ and $H_{k}$ calculated in Equations (4) and (5):

(19) $$O=\left[\begin{array}{c} H_{k}\\ H_{k}F_{k}\\ H_{k}F_{k}^{2}\\ \vdots \\ H_{k}F_{k}^{n-1} \end{array}\right]$$

If this matrix is of full rank, all states are observable. This is a sufficient condition for the estimator Riccati equation to converge to a stable solution [37]. However, this only holds for local observability, while for global observability analysis, the Lie derivatives have to be investigated [36,38]. Due to the non-linear nature of the governing equations, alternative methods need to be implemented to perform observability analysis, such as the ones in [15] or [16]. This work proposes to combine the observability matrices of different timesteps into a large observability matrix. This large observability matrix is a combination of matrices evaluated at evenly spaced timesteps of the training data:

(20) $$O_{tot}=\left[\begin{array}{c} O_{k=1}\\ O_{k=1+p}\\ O_{k=1+2p}\\ \vdots \\ O_{k=m} \end{array}\right]$$

where *k* is the timestep of evaluation, *m* is the total amount of timesteps in the training data and *p* is an integer index that defines the amount of observability matrices that are taken into account in the total observability matrix. The number *p* can be chosen between 1 and *m* depending on the wanted global observability coverage. The smaller the number *p*, the better global observability is analysed, but the larger the total observability matrix. Alternatively, when there are no training data available, the total observability matrix can be calculated for the first timesteps of the simulation based on which the projection can be defined.

Global observability requires the matrix from Equation (20) to be of full rank. However, to reduce costs, installing the minimum amount of sensors needed to produce good and reliable estimation results should always be the aim. Therefore, sensors that do not contribute significantly to the overall quantities of interest estimation performance but are necessary to make all system states observable are not wanted. However, they will cause the estimator to become unstable due to the unboundedness of the unobservable state covariances. This work therefore aims at tackling the stability issues related to unobservable system states. A projection of the Kalman filter Riccati equation is proposed on an observable subspace defined by the singular modes of the complete observability matrix from Equation (20). This projection will be discussed in the next subsection.

### 3.2. Calculation of an Observable Projection Basis

It is well known that a singular value decomposition (SVD) can be used to determine the rank of a matrix [15,39]. Furthermore, an SVD can also indicate which states are unobservable by looking into the modeset corresponding to the singular values. This modeset can also be exploited as a basis for an observable projection.

Lets consider the singular value decomposition of the complete observability matrix. As a result, the full observability matrix ($O_{tot}$) can be written as a product of three matrices:

(21) $$O_{tot}=U\Sigma V$$

where Σ is a square matrix containing the singular values and matrices U and V contain the corresponding modes that satisfy ${\mathrm{VV}}^{T}=I$ and ${\mathrm{UU}}^{T}=I$. When the observability matrix is rank deficient, at least one of the calculated singular values will be close to zero and the matrix Σ will have the following structure:

(22) $$\Sigma =\left[\begin{array}{cccccc} \sigma_{1} & \cdots & 0 & 0 & \cdots & 0\\ \vdots & \ddots & \vdots & \vdots & \ddots & \vdots \\ 0 & \cdots & \sigma_{m} & 0 & \cdots & 0\\ 0 & \cdots & 0 & \approx 0 & \cdots & 0\\ \vdots & \ddots & \vdots & \vdots & \ddots & \vdots \\ 0 & \cdots & 0 & 0 & \cdots & \approx 0 \end{array}\right]=\left[\begin{array}{cc} \Sigma_{o} & 0\\ 0 & \Sigma_{u} \end{array}\right]\approx \left[\begin{array}{cc} \Sigma_{o} & 0\\ 0 & 0 \end{array}\right]$$

where *m* is the rank of the total observability matrix $O_{tot}$. To determine whether singular values are zero, a threshold value of $10^{-12}$ has been set by trial-and-error after investigating the singular values for the validation examples. Furthermore, the corresponding modes present in the matrix V can be partitioned according to the corresponding singular values in Equation (22):

(23) $$V=\left[\begin{array}{c} V_{o}\\ V_{u} \end{array}\right]$$

where $V_{u}$ are the modes that span the kernel of the observability matrix. At the same time, $V_{o}$ is the matrix consisting of the modes corresponding to an observable subspace for this particular sensor set. Using the ${\mathrm{VV}}^{T}=I$ property of the SVD matrices, the following expression can be obtained:

(24) $${\mathrm{VV}}^{T}=\left[\begin{array}{c} V_{o}\\ V_{u} \end{array}\right]\left[\begin{array}{cc} V_{o}^{T} & V_{u}^{T} \end{array}\right]=\left[\begin{array}{cc} V_{o}V_{o}^{T} & V_{o}V_{u}^{T}\\ V_{u}V_{o}^{T} & V_{u}V_{u}^{T} \end{array}\right]=\left[\begin{array}{cc} I & 0\\ 0 & I \end{array}\right]$$

where the $V_{o}V_{o}^{T}=I$ and $V_{o}V_{u}^{T}=V_{u}V_{o}^{T}=0$ properties can be obtained. In this work, a projection of the estimator covariance equations is proposed using the matrix $V_{o}$ containing an observable modeset:

(25) $$x=V_{o}^{T}q$$

The aim of this transformation is to obtain new, observable estimator covariance equations such that the estimator runs stable during operation. Applying Equation (25) to Equations (4) and (5), the new linearised Jacobians can be found as:

(26) $$\begin{array}{ccc} & {\tilde{F}}_{k}=V_{o}F_{k}V_{o}^{T} & \end{array}$$

(27) $$\begin{array}{cc} & {\tilde{H}}_{k}=H_{k}V_{o}^{T} \end{array}$$

Due to the transformation, the new estimator observability matrix will have an additional multiplication by the transformation matrix $V_{o}^{T}$. Using the singular value decomposition formula from Equation (21), Equation (22) and the $V_{u}V_{o}^{T}=0$ and $V_{o}V_{o}^{T}=I$ properties, the following expression is obtained:

(28) $$O{}_{tot}V_{o}^{T}=U\Sigma VV_{o}^{T}=U\left[\begin{array}{cc} \Sigma_{o} & 0\\ 0 & 0 \end{array}\right]\left[\begin{array}{c} V_{o}\\ V_{u} \end{array}\right]V_{o}^{T}=U\left[\begin{array}{cc} \Sigma_{o} & 0\\ 0 & 0 \end{array}\right]\left[\begin{array}{c} V_{o}V_{o}^{T}\\ 0 \end{array}\right]=U\left[\begin{array}{c} \Sigma_{o}\\ 0 \end{array}\right]$$

which proves that the newly obtained observability matrix will be of full rank as all singular values larger than 0 of the original total observability matrix are present in $\Sigma_{o}$.

The above proposed projection only works if the targeted quantities of interest, expressed in the virtual sensor equations through $G_{vs,k}$, are independent of the unobservable states. Mathematically speaking, this means that the linearised Jacobian of the virtual sensors cannot be spun with any of the observability matrix kernel basis vectors $V_{u}$. To check this, the following criterion has to be evaluated:

(29) $$G_{vs,m}V_{u}^{T}=0$$

where *m* indicates that the linearised Jacobian of the quantities of interest equation is taken at the last timestep of the training data to make sure that initial transient behaviour is no longer present.

If the criterion of Equation (29) is not met, the estimator cannot be set up such that it will deliver valuable and trustworthy information on the quantities of interest. If this is the case, the sensor set connected to the Kalman filter should be changed or extended to a set that is able to capture all relevant information.

### 3.3. Transformation of the Estimator Covariance Equations

Now that the projection matrix $V_{o}$ has been defined and the new Jacobians are derived, the new projected Kalman filter equations can be deduced. Using the matrices defined in Equations (9)–(13) and expressing the estimator equations in terms of the linearised Jacobians deduced from Equations (4) and (5), the following equations can be derived:

*Prediction*:

(30) $$\begin{array}{ccc} & x_{k|k+1}=f\left(x_{k},t_{k}\right) & \end{array}$$

(31) $$\begin{array}{ccc} & y_{k+1}=h\left(x_{k},t_{k}\right) & \end{array}$$

(32) $$\begin{array}{ccc} & P_{k|k+1}=\left(V_{o}F_{k}V_{o}^{T}\right)P_{k|k}{\left(V_{o}F_{k}V_{o}^{T}\right)}^{T}+V_{o}Q_{d}V_{o}^{T} & \end{array}$$

*Correction*:

(33) $$\begin{array}{ccc} & S_{k}=\left(H_{k}V_{o}^{T}\right)P_{k|k+1}{\left(H_{k}V_{o}^{T}\right)}^{T}+R & \end{array}$$

(34) $$\begin{array}{ccc} & K_{k}=P_{k|k+1}{\left(H_{k}V_{o}^{T}\right)}^{T}S_{k}^{-1} & \end{array}$$

*Update*:

(35) $$\begin{array}{ccc} & x_{k+1|k+1}=x_{k|k+1}+V_{o}^{T}K_{k}\left(y_{m,k+1}-y_{k+1}\right) & \end{array}$$

(36) $$\begin{array}{ccc} & P_{k+1|k+1}=\left(I-K_{k}\left(H_{k}V_{o}^{T}\right)\right)P_{k|k+1} & \end{array}$$

Finally, the quantities of interest and their corresponding covariances expressed by Equations (17) and (18) can be projected according to the transformation matrix $V_{o}$:

(37) $$\begin{array}{ccc} & y_{vs,k+1}=g\left(x_{k+1|k+1},t_{k}\right) & \end{array}$$

(38) $$\begin{array}{ccc} & P_{vs,k+1}=\left(G_{vs,k}V_{o}^{T}\right)P_{k+1|k+1}{\left(G_{vs,k}V_{o}^{T}\right)}^{T} & \end{array}$$

To acquire the original state covariances, the newly acquired covariances need to be transformed back to their original values. This can be conducted by pre- and postmultiplying the covariance matrix $P_{k+1|k+1}$ with the observable subspace transformation matrix $V_{o}$:

(39) $$\begin{array}{ccc} & P_{k+1|k+1}=V_{o}^{T}P_{k+1|k+1}V_{o} & \end{array}$$

As a result of the transformation, the covariance matrix $P_{k+1|k+1}$ of Equation (36) is no longer of its original size any more but is of size *m* according to Equation (22). This means that, due to the negligible contribution of the unobservable states to the transformation matrix $V_{o}$, these unobservable states will be omitted from the covariance equations, ensuring that their covariances will remain stable during the estimator operation. However, the drawback is that, when transforming the state covariances back to their original values in Equation (39), the corresponding covariances for the unobservable states are not interpretable as the transformation matrix $V_{o}$ does not contain any contributions towards these states. However, if the defined quantities of interest are independent of the unobservable states, their corresponding covariances will be interpretable and deliver reliable results. At the same time, some measurements can be excluded from the estimator, which reduces the need to buy more sensors to solve estimator stability issues related to observability.

## 4. Sensor Selection Using the Stabilised EKF Estimation Framework

The second aspect that is covered in this work is the selection of the appropriate sensors. A sensor selection algorithm is proposed that ranks the sensors according to their contribution to the quantities of interest estimation performance by evaluating the covariance equations based on the training data. The mean of the covariance profile of the quantities of interest is used to evaluate the performance of a certain sensor set.

### 4.1. Generate Training Data by Performing a Forward Simulation

The training data are generated by performing a forward simulation and saving all relevant matrices. This allows evaluating the sensor performance beforehand, hence eliminating the need for an initial measurement campaign on a physical system and the acquisition of sensors.

During the forward simulation, the relevant information needed for further processing is stored. All sensors to be included in the sensor selection algorithm should be present in the training data, such that the generated linearised Jacobian for the measurement equation $H_{k}^{n_{s}^{0}}\in R^{\left(n_{s}^{0}\right)\times \left(n_{st}\right)}$ has the correct dimensions corresponding to the total amount of sensors in the sensor space $n_{s}^{0}$.

### 4.2. Sensor Selection Algorithm

Very often in state estimation, it is not clear which sensors contribute the most towards the estimation performance. Therefore, the proposed sensor selection algorithm aims at ranking the sensors by their estimation performance, which is evaluated using the quantities of interest covariance matrices $P_{vs,k}$ for each simulation timestep. During the sensor selection procedure, no state update is performed, but only the covariance propagation is evaluated. This allows the algorithm to be sped up significantly. The linearised Jacobian matrices are reused from the training data generated according to the previous subsection. The sensor selection algorithm consists of the following steps (where $n_{s}^{0}$ is the number of sensors in the entire sensorspace):

1. Given the measurement matrix $H_{k}^{n_{s}^{0}-i}$ for the entire sensor space, and previously saved matrices $F_{k}$ from the training data, evaluate the covariance propagation $P_{vs,k}$ for all simulation timesteps. The resulting mean of the quantity of interest covariance profile is the reference covariance $p_{0}$.
2. Iterate along the entire sensor space and perform during each iteration *i*:
  (a) remove a sensor *j* from the sensor space obtaining $H_{k}^{n_{s}^{0}-i-j}$;
  (b) calculate transformation matrix $V_{o}$ and observability matrix kernel $V_{u}$;
  (c) check that none of the selected sensors depend on the kernel of the observability matrix $G_{vs,m}V_{u}=0$. If any of the selected sensors are part of the kernel, the algorithm is stopped as the sensors are incapable of accurately estimating the quantities of interest;
  (d) if the previous criterion is fulfilled, propagate Kalman covariance equations defined by Equations (32)–(38) from Section 3.3 to obtain $P_{vs,i,k}$. The resulting mean of the quantity of interest is the sensor set covariance $p_{i}$.
3. Find the sensor with the lowest covariance $p_{i}$, remove it from the sensor space and restart Step 2.

The result of the algorithm is a set of sensors ranked by their respective performance contribution to the overall quantity of interest covariance. The algorithm is summarised in Algorithm 1.
**Algorithm 1** Sensor selection1:Given $H_{k}^{n_{s}^{0}-i}$, simulate covariance propagation to obtain reference covariance $p_{0}$2:**for** $i=1:n_{s}^{0}$**do**3:    **for** $j=1:n_{s}^{0}-i+1$ **do**4:          Remove sensor j from sensor space to obtain $H_{k}^{n_{s}^{0}-i-j}$5:          Calculate transformation matrices $V_{o}$ and $V_{u}$ as defined in Section 3.26:          **if** $G_{vs,m}V_{u}=0$ **then**7:                Simulate covariance propagation8:                Evaluate mean of quantity of interest covariance profile:9:                $p_{i}=mean\left(P_{vs,k}\;\forall \;k\right)$10:          **else**11:               Quantities of interest lie within the kernel of observability matrix12:               Stop algorithm;13:    Evaluate the sensor *f* with lowest covariance for the quantity of interest:14:    $f=min(p_{i})$15:    Save sensor *f* and remove *f* from sensor space to obtain $H_{k}^{n_{s}^{0}-j-1}$

## 5. Validation and Discussion

In this work, two validation cases are presented:

1. A simple, numerical example to show the potential of the proposed methods to stabilise the estimator for unobservable states;
2. An experimental validation case to show the engineering cases.

These validation cases are discussed in the next sections.

### 5.1. Simple Validation Case

**Figure 1:**
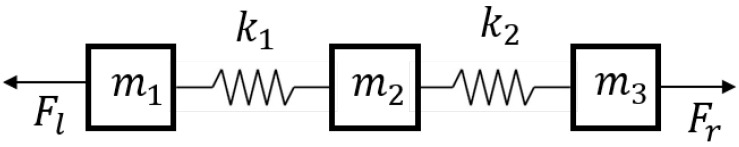
A visual representation of the academic validation case used consisting of three masses connected with springs.

The simple validation case presented in this work is based on a three-mass system depicted in Figure 1.

In this case, the quantity of interest is the position of the left mass $m_{1}$, whereas the measurement used is the position of mass $m_{2}$.

#### 5.1.1. Model Setup

For this simple example, the parameters $k_{1}$ and $k_{2}$ are chosen to be the same, such that $k_{1}=k_{2}=k$. The same holds for the masses $m_{1}=m_{2}=m_{3}=m$. This is conducted to simplify the system and make the results more intuitive. All system parameters are listed in Table 1.

Depending on variables $k_{1}$, $k_{2}$ and the masses of the blocks, three eigenmodes can be computed for this system. For this example, the three eigenfrequencies are 0, km and 3km. The system is excited with a sinusoidal force at the exact frequency of the second eigenmode, being km=100Hz. Corresponding to this eigenfrequency, the eigenmode is of the form:

(40) $$V_{2}={\left[\begin{array}{ccc} -1 & 0 & 1 \end{array}\right]}^{T}$$

This is the mode where both outer masses move in opposite directions, and the middle mass does not move. This means that the middle mass $m_{2}$ is located in a node of this eigenmode. Therefore, a position measurement of this mass cannot provide any information on the states of both other masses, and hence, the estimator will be unobservable. Here, the proposed projection can be executed to stabilise the Kalman filter covariances.

#### 5.1.2. Estimator Setup

To estimate the quantities of interest, the filter has to be configured with a model noise matrix $Q_{d}$ and a measurement noise matrix R. For the model noise matrix $Q_{d}$, a noise level of (10${}^{-2}$ m${}^{2}$) has been set for the acceleration equations, whereas the noise level for the velocity equations are being set to 0. This is due to the fact that the velocity equations are a copy from the previous timestep, and therefore, no error is introduced. This means that the $Q_{d}$-matrix is defined as follows:

(41) $$Q_{d}=\left[\begin{array}{cccc} 10^{-2} & 0 & 0 & 0_{1\times 3}\\ 0 & 10^{-2} & 0 & 0_{1\times 3}\\ 0 & 0 & 10^{-2} & 0_{1\times 3}\\ 0_{3\times 1} & 0_{3\times 1} & 0_{3\times 1} & 0_{3\times 3} \end{array}\right]$$

The measurement noise is uncorrelated with a variance of ( 2 × 10${}^{-1}$ (m/s${}^{2}$)${}^{2}$) for acceleration measurements and/or (10${}^{-1}$ m${}^{2}$) for position measurements.

#### 5.1.3. Discussion

This academic example allows showing that the projection onto the observable subspace can stabilise the state covariances of the Kalman filter. To show this, a special case is selected where the system is excited on exactly the second eigenmode, and a position measurement of mass $m_{2}$ is considered. The quantity of interest is the acceleration of mass $m_{1}$. Since the system is excited on an eigenfrequency, a position measurement of mass $m_{2}$ will not deliver any useful data to track the acceleration of mass $m_{1}$ as this mass lies within a node of the second eigenmode of the system. This special case is chosen to show that the proposed methodology works for any system where the unobservable modes can be isolated using a singular value decomposition.

**Figure 2:**
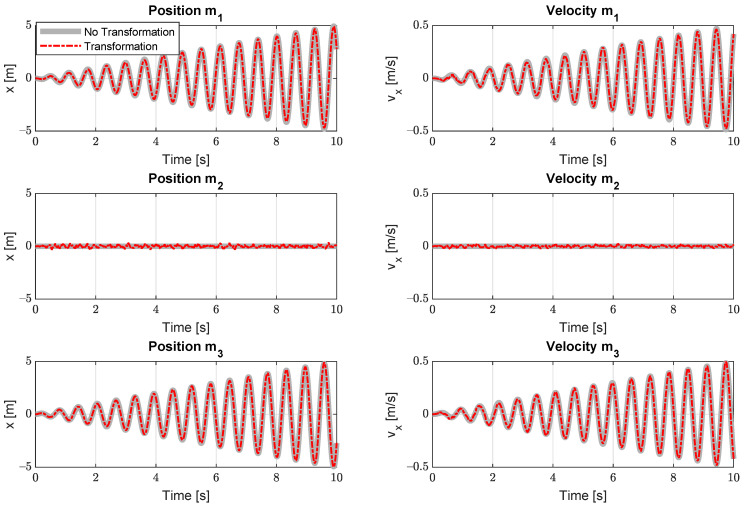
State estimation results for the academic model. The graphs shows the difference between the estimation results with covariance transformation versus without.

The results of the estimation are depicted in Figure 2 and are similar to the results obtained from other simple, academic example cases [35]. One can observe that the amplitude of the oscillations raises continuously due to the system excitation on resonance frequency and the lack of damping in the model. The estimator is able to track the states of the system well, although larger variations in the position of mass $m_{2}$ can be observed where the absolute state values reach 0 m. Because this mass is not moving much, it becomes harder for the estimator to track.

**Figure 3:**
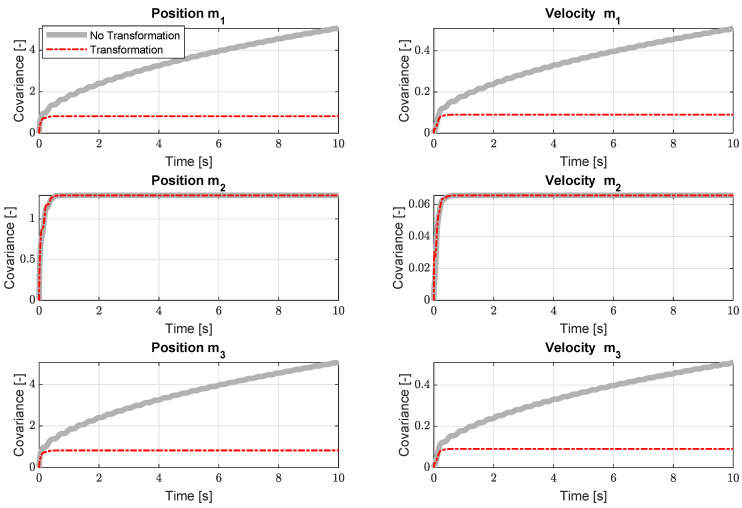
The resulting state covariances for the academic model using a position measurement on the middle mass $m_{2}$ and a virtual position measurement of mass $m_{3}$.

For this system, a position measurement on mass $m_{2}$ will cause the states of both the outer masses $m_{1}$ and $m_{3}$ to be unobservable. This is visible in Figure 3 due to the unboundedness of the covariances of the states corresponding to masses $m_{1}$ and $m_{3}$ shown in grey as they are linked to the kernel of the observability matrix $V_{u}$.

Using the projection shown in Section 3, these unstable covariances can be stabilised. When applying the projection, the resulting covariances are bounded, which is indicated by the red dashed lines in Figure 3. Figure 3 also shows that, since the states of the middle mass $m_{2}$ were already fully observable, the projection does not influence these covariances. However, one has to be careful interpreting the covariances of any quantity of interest should they be linked to an unobservable modeset $V_{u}$, which can be investigated using Equation (29). Because the unobservable system states remain unobservable when applying the projection, the estimation results of these states should be interpreted carefully. This indicates that the transformation introduces a trade-off between the stability of the Kalman filter and the interpretability of the covariances. When this would be the case in practise, additional sensors will have to be added to the sensor set in order to comply with the observability requirements. Nevertheless, the results show that the proposed methodology is able to stabilise the unobservable covariances, hence ensuring a stable estimator containing unobservable states.

### 5.2. Experimental Validation Case

The second validation case for the proposed estimator approach is an experimental case performed on a vehicle. The experimental validation is conducted on a vehicle using a 10-DOF vehicle model that has been developed and validated at Flanders Make in previous work [1,40,41] and graciously provided to be used in this work.

#### 5.2.1. Model Setup

**Figure 4:**
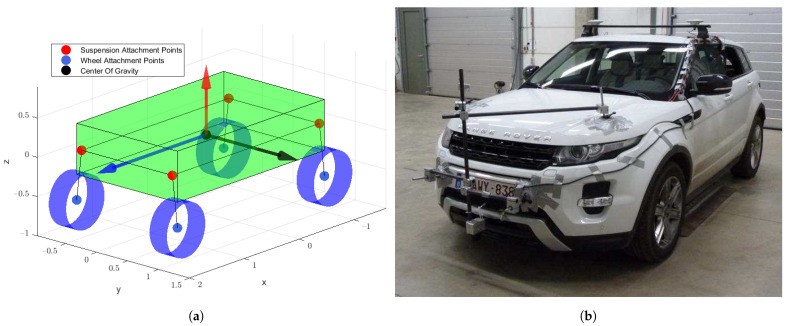
An overview of the vehicle application case for the validation of this work. (**a**) The 10-DOF model used for experimental validation. (**b**) The Flanders Make Range Rover Evoque represented by the 10 DOF model [1].

The model used in this work is a 10-DOF freedom model is depicted in Figure 4, which is based on the Flanders Make Range Rover Evoque, visible in the same figure. The model has 16 states in total, 12 states for the body (2 for each degree of freedom) and 4 to describe the rotational degree of freedom of the wheels. Geometric parameters have been measured on the real vehicle, such as the suspension attachment points, centre of gravity location, wheel centre locations and so on. Table 2 gives an overview of the main parameters of the Range Rover Evoque, which are used by the 10-DOF vehicle model.

Next to these general body definitions, the-10 DOF vehicle models features:

- An exponential suspension spring characteristic;
- A linear suspension damper model;
- A linear tire model using cornering stiffnesses from Table 2.

The measurements were generated during a test campaign at Ford Lommel [40]. Here, the vehicle has been equipped with various sensors to measure the dynamic quantities of the system, among which a low-cost Global Navigation Satellite System receiver, an inertial measurement unit (IMU), a Corrsys Datron optical sensor and Kistler tire force transducers [1]. The IMU measured the accelerations in three directions of the centre of gravity and rotational speeds and angles (roll, pitch and yaw), defined around the axes depicted in Figure 4. The Corrsys Datron optical sensor measured the longitudinal and lateral velocity of the car, of which the slip angle could be deduced. The GPS was able to record the location of the vehicle’s centre of gravity during the measurements, and the tire force transducers measured the tire forces of both rear tires.

For this validation case, several quantities of interest have been investigated. These considered metrics are listed below:

1. Rear longitudinal tire forces;
2. Rear lateral tire forces;
3. Rear vertical tire forces;
4. Vehicle longitudinal and lateral position;
5. Vehicle side-slip angle.

The tire forces were estimated as this is commonly conducted already since tire force transducers are very expensive. The side-slip angle is also a subject of current research as this variable is hard to measure due to its dynamic nature but necessary for advanced control algorithms such as the ESP. Finally, the vehicle longitudinal and lateral position states were also included in the quantities of interest to show that the proposed projection and sensor selection methodology work for independent states.

#### 5.2.2. Estimator Covariance Tuning

A necessary step to set up any estimator is to tune the covariance matrices. The model covariance matrix $Q_{d}$ used for this validation case has been derived as:

(42) $$Q_{d}=\left[\begin{array}{ccccc} 1\times 10^{-4}I_{3\times 3} & 0_{3\times 3} & 0_{3\times 3} & 0_{3\times 3} & 0_{3\times 4}\\ 0_{3\times 3} & 1\times 10^{-3}I_{3\times 3} & 0_{3\times 3} & 0_{3\times 3} & 0_{3\times 4}\\ 0_{3\times 3} & 0_{3\times 3} & 1\times 10^{-5}I_{3\times 3} & 0_{3\times 3} & 0_{3\times 4}\\ 0_{3\times 3} & 0_{3\times 3} & 0_{3\times 3} & 1\times 10^{-1}I_{3\times 3} & 0_{3\times 4}\\ 0_{4\times 3} & 0_{4\times 3} & 0_{4\times 3} & 0_{4\times 3} & 1\times 10^{-5}I_{4\times 4} \end{array}\right]$$

The matrix defined above has been tuned by trial and error by means of evaluating the covariance values of the estimator.

The measurement noise matrix R is defined based on data sheets of the sensor manufacturer and a set of reference measurements [1]. The used noise levels are listed below:

$$\begin{array}{cc} & R_{a_{i}}=3.1\times 10^{-3}\;{(m/s{}^{2})}^{2},R_{gyr}=2.491\times 10^{-1}\;{(m/s)}^{2}\\ & R_{GPS}=2\;m^{2},R_{v_{i}}=4.856\times 10^{-2}\;{(m/s)}^{2},R_{\omega_{ij}}=2.1\times 10^{-1}\;{\left(m/s\right)}^{2} \end{array}$$

#### 5.2.3. Discussion

**Figure 5:**
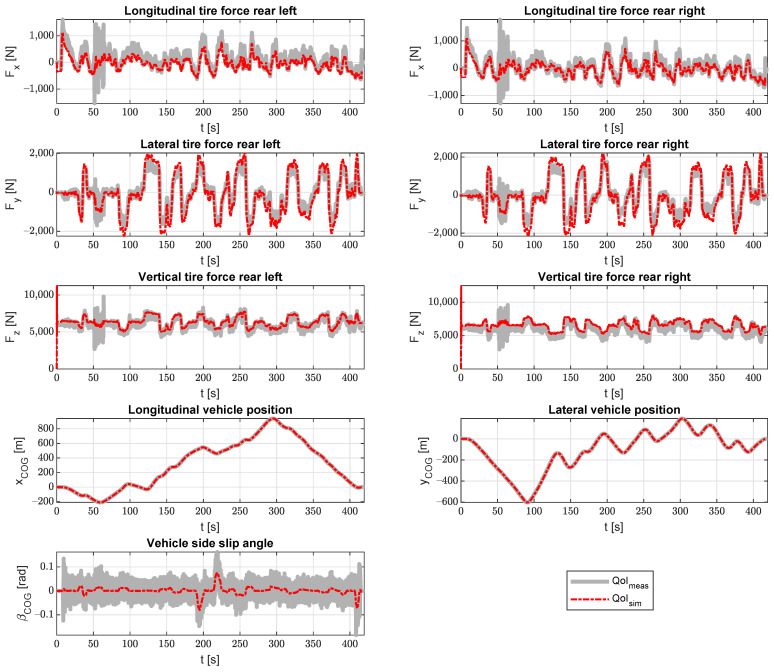
Validation of the quantities of interest (QoI) using real measurements from the Flanders Make Range Rover Evoque.

Figure 5 shows the results of the estimator compared to the measurements from the Range Rover Evoque. The quantities of interest, as listed in the section above, are compared to the measurement data from the experimental campaign. The sensors used in the Kalman filter were the IMU accelerations in three directions, a gyroscope for the yaw rate measurement, the GPS (vehicle *x* and *y*) and wheel speed sensors.

In general, the results match very well. One thing that can be noted is that the lateral forces are a bit over estimated in the extreme regions. Similar results were observed in [40] for the accelerations and the yaw rate when a linear tire model was used. This can be explained by the use of a linear tire model that does not take into account the saturation of the tire. This causes the forces generated by the tire to be larger than the ones actually measured. The use of a more complex, non-linear tire model that can take tire saturation into account will fix this issue and can be addressed in future work.

**Figure 6:**
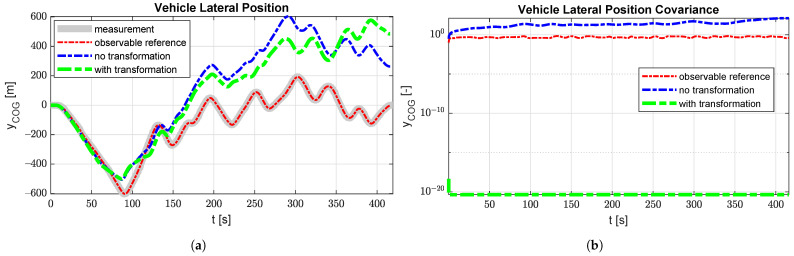
Comparison of the vehicle lateral position state for an observable case, an unobservable case without projection and an unobservable case with projection. (**a**) The vehicle lateral position state compared to the measured GPS Signal. (**b**) The covariance corresponding to the lateral vehicle position state.

This validation case can show the potential of the covariance stabilisation methodology presented in this work on an engineering case as vehicle absolute position states are typically dynamically independent from the rest of the vehicle states when the road is considered flat. Therefore, if the tire forces are targeted by the estimator, the GPS measurements are less relevant for the estimator performance but will immediately cause the position states to be unobservable. This is depicted in Figure 6b where the blue covariance curve is rising continuously throughout the simulation. The green covariance curve on the same graph shows that the transformation can stabilise the covariance as it is now bounded, but orders of magnitude lower than for the observable case (red line). However, Figure 6a shows that the position is not tracked well during the simulation for all unobservable cases causing the vehicle position results to be poor. This indicates that one should be careful when interpreting the resulting covariance when using the projection for unobservable states. The proposed projecion therefore introduces a trade-off between the stability of the estimator and the interpretability of the results. However, if only interested in some specific quantities of interest, this approach can significantly reduce the cost of deploying extra sensors to obtain full observability, especially for applications with lots of states [6].

**Figure 7:**
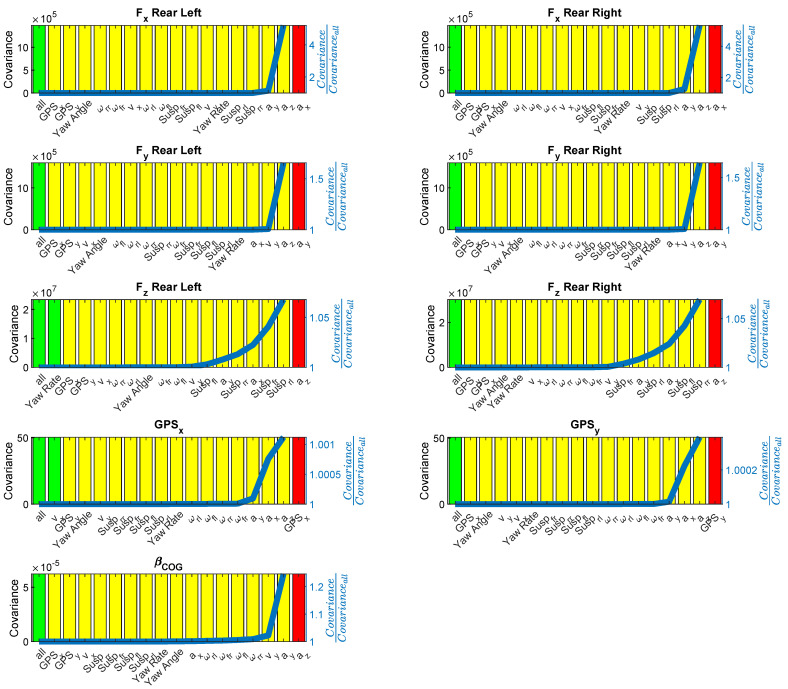
The sensor selection methodology results. For each quantity of interest, the sensor space has been reduced from left to right. The observability is indicated with the colour (green is fully observable, yellow is unobservable but stable and red is unobservable). The normalised metric covariance is indicated by the blue line.

The second part of the validation is to select the appropriate sensors for the estimation of the different quantities of interest that are considered. This is conducted by ranking the sensors based on their relative contributions to the covariance of the quantity of interest, similar to the approaches used in [26,27]. Figure 7 shows the results of the approach presented in Section 4. Figure 7 shows the contribution of each sensor to the quantities of interest covariance ($p_{0}$) relative to a simulation, including the complete sensor set (indicated by the blue line) at each iteration of Algorithm 1 where the sensor set has been reduced by exactly one sensor from left to right. Furthermore, the colour of the bars indicate information on observability. A green bar indicates that the observability is fulfilled during the entire simulation. A yellow bar indicates that the projection of Section 3 was carried out that stabilised the unobservable state covariances, and a red bar indicates that it is impossible to estimate the quantities of interest with this sensor set.

In this validation case, only the last iteration is marked with a red colour, indicating that, at the least, this sensor is needed to provide the necessary data to comply with the observability requirements. Additionally, the blue line indicates that, for most quantities of interest within this validation example, only a few sensors contribute significantly to the overall quantity of interest covariance. The rest of the sensors have negligible added value relative to the quantities of interest performance.

Figure 7 shows that for the tire forces, the acceleration measurements in the respective directions are the most important sensors as they are directly related to the forces in the equations of motion. Adding these sensors can increase the estimator performance by a factor of 5, as indicated by the blue line on Figure 7. One can also observe that GPS measurements are of lesser importance here and are situated on the left side of the graph. However, omitting these GPS measurements causes the vehicle position states to be unobservable (as indicated by the yellow bar colour), which can be solved by applying the transformation proposed in Section 3.

On the other hand, when trying to evaluate the position states of the vehicle, the results depicted in Figure 7 show that not much performance can be gained by adding additional sensors as these states have no dependency to other states because they are directly obtained from integration. This means that GPS measurements are absolutely necessary to be able to track these states. The proposed approach is able to identify the situations where the estimator cannot be set up using the current sensor set by evaluating Equation (29).

In addition to the approaches presented in [25,26,27], the approach proposed in this work is able give a sensor performance indication for all types of sensors while also being able to handle the same type of sensors on different mounting locations as well as different sensor noise levels (cheap versus expensive sensors). Of course, the results of the sensor selection can be significantly different when considering multiple quantities of interest at the same time. This has not been investigated in this work and can be addressed in future work.

## 6. Conclusions

In this work, an algorithm is proposed to stabilise estimators for unobservable system states, given that only specific quantities of interest are targeted by the estimator. Through a projection of the estimator on an observable subspace, the employed Riccati equations can be stabilised, but care needs to be taken as this does not make the system inherently observable. To ensure reliable operation, the targeted quantities of interest should not rely on the non-observable omitted system subspace. This approach has been validated on a numeric academic mass-spring system, as well as an industrial automotive estimation problem. Compared to known solutions, this method is shown to consistently handle cases where decoupled states are present, strongly coupled modes are present, as well as complex non-linear behaviour.

Next to the estimation stabilisation approach, a sensor selection algorithm was proposed that ranks the sensor performance based on the expected uncertainty on the various quantities of interest. Starting form an exhaustive potential sensor set, the ranking procedure is performed by gradually removing one sensor at a time, which induces the lowest covariance rise when omitted from the estimator. On the automotive validation case, the method automatically detects that for tire forces, the acceleration measurements coming from the IMU are the most important sensors, whereas for the vehicle position states, the GPS measurements would be the most effective, which is consistent with engineering experience. Therefore, compared to known solutions, the approach is able to provide insight into the sensor set performance with respect to the predefined quantities of interest without the need for acquiring sensors and setting up costly test campaigns and is able to detect when a particular sensor set is unable to bring sufficient data in an estimator context.

## Figures and Tables

**Table 1:** Parameters of the three-mass academic example system used for validation.

Parameter	Value
$m_{1}$, $m_{2}$, $m_{3}$ (kg)	10
$k_{1}$, $k_{2}$ (N/m)	1000

**Table 2:** Vehicle model parameters for the Range Rover Evoque [1,40].

Vehicle Property	Abbreviation	Value
Vehicle mass	*m*	2408 kg
Yaw moment of inertia	$I_{zz}$	$3231\;{\mathrm{kgm}}^{2}$
Distance between COG and front axle	$l_{f}$	1.4394 m
Distance between COG and rear axle	$l_{r}$	1.2356 m
Track width of front axle	$t_{f}$	1.625 m
Track width of rear axle	$t_{r}$	1.625 m
Height of COG	$h_{COG}$	0.65 m
Front axle cornering stiffness	${\bar{C}}_{yf}$	88,500 N/rad
Rear axle cornering stiffness	${\bar{C}}_{yr}$	118,200 N/rad

## Data Availability

The data presented within this study are resulting from activities within the acknowledged projects and are available therein.

## Abbreviations

The following abbreviations are used in this manuscript:

GPS	Global Positioning System
SVD	Singular Value Decomposition
EKF	Extended Kalman Filter
FIM	Fisher Information Matrix
(D)ARE	(Discrete) Algebraic Riccati Equation
DOF	Degree(s) Of Freedom
IMU	Inertial Measurement Unit
ESP	Electronic Stability Program
QoI	Quantities of Interest
